# Fibrin glue does not assist migration and proliferation of chondrocytes in collagenic membranes: an in vitro study

**DOI:** 10.1186/s13018-022-03201-6

**Published:** 2022-06-11

**Authors:** Filippo Migliorini, Julia Prinz, Nicola Maffulli, Jörg Eschweiler, Christian Weber, Sophie Lecoutrier, Frank Hildebrand, Johannes Greven, Hanno Schenker

**Affiliations:** 1 Department of Orthopaedic, Trauma, and Reconstructive Surgery, RWTH University Hospital, Pauwelsstraße 30, 52074 Aachen, Germany; 2 Department of Ophthalmology, RWTH University Hospital, Pauwelsstr. 30, 52074 Aachen, Germany; 3 Department of Medicine, Surgery and Dentistry, University of Salerno, 84081 Baronissi, SA Italy; 4 School of Pharmacy and Bioengineering, Faculty of Medicine, Keele University, ST4 7QB Stoke on Trent, England; 5 Barts and the London School of Medicine and Dentistry, Centre for Sports and Exercise Medicine, Queen Mary University of London, Mile End Hospital, 275 Bancroft Road, E1 4DG London, England

**Keywords:** Chondral defects, Autologous chondrocyte implantation, Fibrin

## Abstract

**Background:**

Some authors secured the membrane during matrix-induced autologous chondrocyte implantation (mACI) with fibrin glue or did not use a formal fixation. The real impact of fibrin glue addition on chondrocytes migration and proliferation has not yet been clarified. This study evaluated the impact of fibrin glue on a chondrocyte loaded collagenic membrane.

**Methods:**

A resorbable collagen I/III porcine derived membrane commonly employed in AMIC was used for all experiments. Chondrocytes from three difference donors were used. At 1-, 2-, 3-, 4-, 6-, and at 8-week the membranes were embedded in Mounting Medium with Dapi (ABCAM, Cambridge, UK). The Dapi contained in the mounting medium ties the DNA of the cell nucleus and emits a blue fluorescence. In this way, the spreading of the cells in the membrane can be easily monitored. The outcomes of interest were to evaluate (1) cell migration and (2) cell proliferation within the porous membrane layer. DAPI/nuclei signals were analysed with fluorescence microscope under a magnification of 100-fold.

**Results:**

The no-fibrin group demonstrated greater migration of the cells within the membrane. Although migration resulted higher in the no-fibrin group at every follow-up, this difference was significant only at week 1 (*P* < 0.001), 2 (*P* = 0.004), and 3 (*P* = 0.03). No difference was found at week 3, 6, and 8. The no-fibrin group demonstrated greater proliferation of the chondrocytes within the membrane. These differences were significant at week 4 (*P* < 0.0001), 6 (*P* < 0.0001), 8 (*P* < 0.0001).

**Conclusion:**

The use of fibrin glue over a resorbable membrane leads to lower in vitro proliferation and migration of chondrocytes.

## Introduction

Matrix-induced autologous chondrocyte implantation (mACI) has been advocated inpatients with symptomatic chondral defects unresponsive to conservative management [1, 2]. During mACI, autologous chondrocytes are harvested from a non-weight bearing zone of the knee, and expanded over a bioresorbable membrane in an external laboratory [3, 4]. The chondrocyte-loaded membrane is subsequently implanted into the defect in a second surgical session [5, 6]. How such membrane is secured into the defects vary. Initially, the membrane was sutured to the defect to ensure implant stability. However, suture generates partial-thickness lesions of the articular cartilage which may not heal and enlarge with time, leading to persisting symptoms and premature degeneration [7–9]. To avoid membrane suture, fibrin glue has been introduced, although some studies secured the membrane with suture [10, 11], fibrin glue [12–21], or both [22–25]. However, the membrane remains stable in the defects even without formal fixation [26–30]. Such heterogeneity in membrane fixation arises from the limited evidence and lack of consensus. The real impact of fibrin glue addition on chondrocytes migration and proliferation has not yet been clarified. This study evaluated the impact of fibrin glue on a chondrocyte loaded collagenic membrane. An in vitro study was conducted to evaluate chondrocyte migration and proliferation with or without fibrin glue application in a porcine derived collagen I/III membrane commonly employed in mACI.

## Methods

### Study protocol

**Fig. 1:**
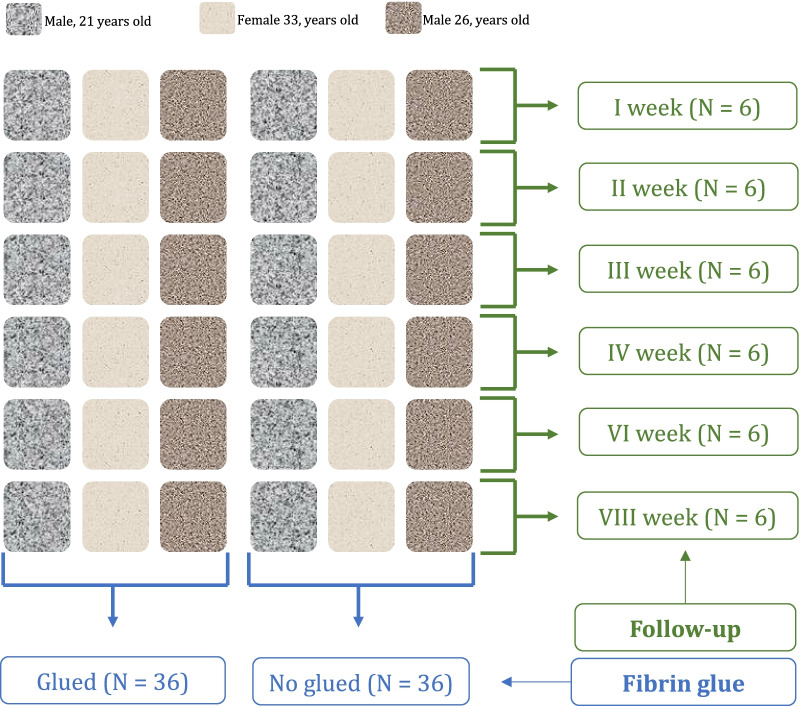
Experimental set-up (*N* = 72)

The present study was approved by the ethical committee of the Medical Faculty of the University RWTH of Aachen (ID EK305-13). A resorbable collagen I/III porcine derived membrane (Cartmaix, Matricel GmbH, Herzogenrath, Germany) commonly used in mACI was used for all experiments. Each experiment was repeated three times each containing different donor cells: a 35 years old male, a 34 years old male, and a 21 years old female. The membranes were cut into 0.7 × 0.7 cm (area 0.49 cm^2^) in a sterile fashion. Overall, 72 membranes were used for the experiments: 36 non-glued and 36 membranes with fibrin glue (Tisseel, Baxter International Inc, Illinois, USA). Cell proliferation and migration were compared at 1-, 2-, 3-, 4-, 6-, and at 8-week follow-up. This process is schematised in Fig. 1.

### Chondrocyte processing

Each membrane was seeded with chondrocytes on the porous side. Following trypsinization (Sigma-Aldrich/Merck KGaA, Darmstadt, Germany) and centrifugation (1500 rmp, 10 min), the chondrocytes were resuspended in a volume of 40 µl per membrane, and spread as homogenous as possible over the membranes of a density per membrane of approximately 100,000 MSCs per cm^2^. After cultivation of 2 h at 37 °C in the incubator, the wells were filled up with cell culture medium. The cell culture medium was composed as follow: Dulbecco's Modified Eagle’s Medium (DMEM) combined with 4,5 g/l D-Glucose (GlutaMax, high glucose, Gibco/Life Technologies, Paisley, UK), 10% fetal calf serum (FCS, Pan-Biotech, Aidenbach, Germany), 1% penicillin–streptomycin (Pen/Strep, Sigma-Aldrich/Merck KGaA, Darmstadt, Germany). The medium was changed every 3 days.

### Experiments

At 1-, 2-, 3-, 4-, 6-, and at 8-week follow-up a membrane was fixed in 4% paraformaldehyde (Merck Schuchardt OHG, Hohenbrunn, Germany) for 12 h. Afterwards, the membranes were dehydrated in an ascending alcohol series (5 min per cuvette) as follow: xylene (3×), 100% ethanol (2×), 96% ethanol, 80% ethanol, 70% ethanol, aqua dest. Subsequently the membranes were embedded in paraffin (Sakura Finetek Europe B.V., Alphen aan den Rijn, Netherlands) and cooled to − 10 °C. 3 µm sized cuts were prepared on a microtome (Schlittenmikrotom PFM Slide 4003E, PFM Medical AG, Cologne, Germany). To allow better adherence on the specimen slides, the cuts were heated at 60 °C for an hour. The paraffin of the slices was removed with xylol (Otto Fischar GmbH&Co KG, Saarbrücken, Germany) and afterwards the slices were carefully rehydrated with a descending alcohol series as follow: xylene (3X), 100% ethanol (2×), 96% ethanol, 80% ethanol, 70% ethanol, aqua dest. The membranes were embedded in Mounting Medium with Dapi (ABCAM, Cambridge, UK) and photographed on the fluorescence microscope (DM/RX, Leica, Wetzlar, Germany). The Dapi contained in the mounting medium ties the DNA of the cell nucleus and emits a blue fluorescence, allowing to detect how the cells in the membrane have spread.

### Outcomes of interest

The outcomes of interest were (1) to evaluate cell migration and (2) cell proliferation within the porous membrane layer. DAPI/nuclei signals were analysed with fluorescence microscope at 100-fold magnification and the software Image J version 1.51 (National Institutes of Health, US). Migration was expressed as the percent of ingrowth of such cell within the overall thickness of the membrane. The cells which migrated in the deepest layer of the membrane was used a reference. Proliferation refers to the number of cells per mm^3^.

### Statistics

All statistical analyses were performed using the IBM SPSS Statistics version 28.0 (IBM Corporation, Armonk NY, USA). The Shapiro–Wilk test was performed to investigate data distribution. For normally distributed variables, the *t*-test (Welch) was used, and the Mann Whitney U test was used for non-parametric data.

## Results

### Migration

**Fig. 2:**
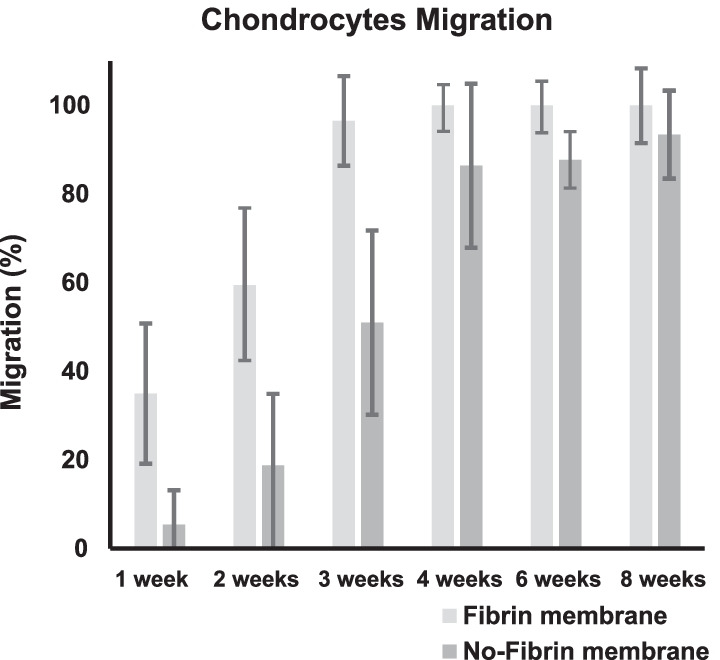
Chondrocytes migration within the membrane

The no-fibrin group demonstrated greater migration of the cells within the membrane. Although migration resulted higher in the no-fibrin group at every follow-up, this difference was significant only at week 1 (*P* < 0.001), 2 (*P* = 0.004), and 3 (*P* = 0.03). No difference was found at week 3, 6, and 8. Figure 2 shows the results of cell migration at each follow-up.

### Proliferation

**Fig. 3:**
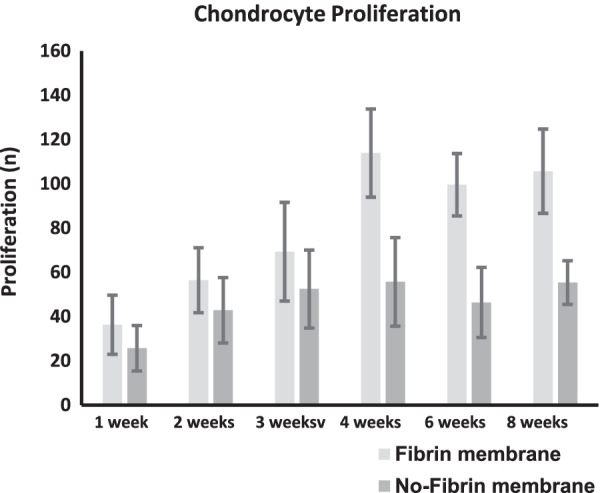
Chondrocytes proliferation within the membrane

The no-fibrin group demonstrated greater proliferation of the chondrocytes within the membrane. These differences were significant at week 4 (*P* < 0.0001), 6 (*P* < 0.0001), 8 (*P* < 0.0001). Figure 3 shows the results of cell proliferation at each follow-up.

## Discussion

**Fig. 4:**
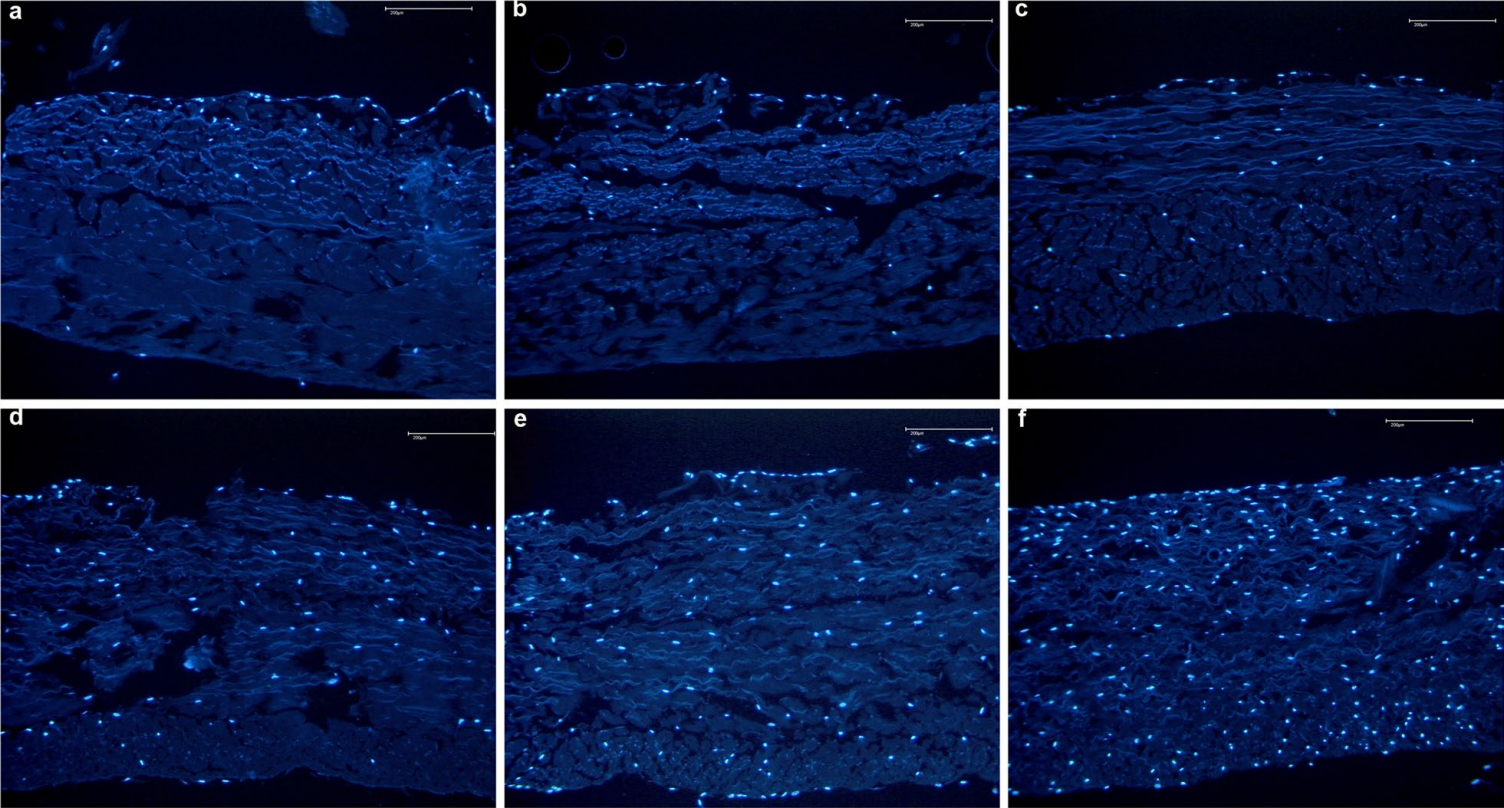
Proliferation and migration of chondrocytes in the membrane without fibrin using the DAPI stained cell nuclei of chondrocytes (blue). Time dependent proliferation and migration of chondrocytes into the non-fibrin treated membrane over the 1, 2, 3, 4, 6, and 8 weeks (picture **a–f**, respectively)

**Fig. 5:**
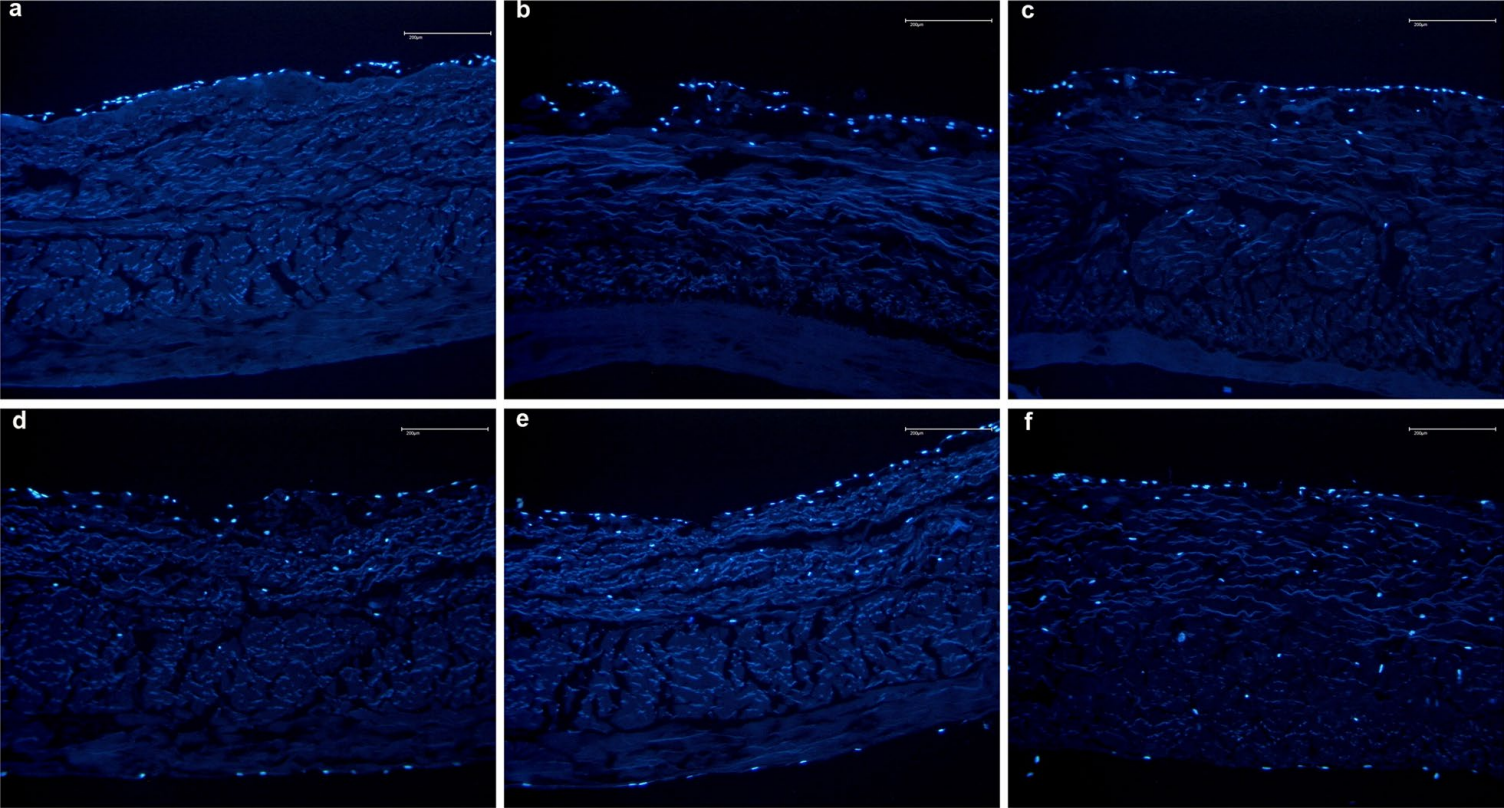
Proliferation and migration of chondrocytes in the fibrin glued membrane using the DAPI stained cell nuclei of chondrocytes (blue). Time dependent proliferation and migration of chondrocytes into the non-fibrin treated membrane over the 1, 2, 3, 4, 6, and 8 weeks (picture **a–f**, respectively)

According to the main findings of the present study, the use of fibrin glue over a resorbable membrane leads to lower in vitro proliferation and migration of chondrocytes (Figs. 4 and 5). The membranes without fibrin glue demonstrated greater chondrocytes migration of the cells within the first three weeks, and greater proliferation during the last five weeks.

Initially, it was believed that such membranes should be fixed using sutures in autologous chondrocyte implantation. However, studies demonstrated that suture can irreversibly damage the cartilage. Suturing the membrane generates partial-thickness lesions of the articular cartilage which may not heal and enlarge with time [7, 8]. Hunzinker et al. [9], to establish the potential damage of sutures in cartilage, sutured the surrounding articular cartilage of large, partial-thickness trochlear defects in 18 adult goats. The perisutural area underwent histological, histochemical and histomorphometrical analysis: suturing induced severe local cartilage impairment which may lead to pain, reduced healing and premature osteoarthritis [9]. To avoid this, membrane suture fibrin glue has been introduced. Fibrin glue has been widely employed given its biological sealing, haemostatic and adhesive proprieties [31, 32]. Its primary use is as a biological sealant, and it also promotes chondrocytes migration and proliferation [33–35]. Mainly through the action of thrombin, fibrin glue is believed to promote a variety of cellular responses, increasing cell migration, proliferation and survival [36–40].

The success of chondrocyte cultivation in a matrix depends on the ability of the implanted chondrocytes to proliferate and synthesize cartilage matrix [41]. For cartilage, platelet derivates such as fibrin directly induce regeneration of the injured tissue [42], but also provide a scaffold carrying biochemical stimuli [43]. The ideal injectable hydrogel for neocartilage formation would be non-reactive, biocompatible, biodegradable at an appropriate rate, and able to support growth factor delivery [44, 45]. To date, numerous natural and synthetic biomaterials have been used for neocartilage tissue engineering, including chitosan [46], collagen/gelatin [47], alginate [48], and fibrin [49].

Fibrin glue is a biological tissue adhesive imitating the final stages of the blood coagulation cascade [50]. Fibrin glue contains a fibrinogen component and a thrombin component which are prepared by processing blood plasma [51]. Previous studies demonstrated fibrin glue to provide mitogenic and chemotactic stimuli for mesenchymal stem cells by releasing platelet-derived growth factor, which promotes cell proliferation, migration, and matrix synthesis [52]. Therefore, fibrin glue has been suggested to sustain isolated chondrocytes, promoting cell proliferation and matrix synthesis [53]. Fibrin glue has been shown to affect extracellular matrix formation, accumulation of collagen, and increase of spherical-shaped chondrocytes in vitro [41]. Commercially available fibrin glue has been applied in clinical practice for some decades [54]. However, fibrinolysis in vivo leads to rapid resorption of the fibrin glue [54]. In 2007, Eyrich et al. [55] developed a long-stable fibrin glue by a modification of buffer substances and supplementation of antifibrinolytic agents. Their fibrin glue hydrogel has been used for tissue engineering of cartilaginous tissues with cultured chondrocytes [56]. Homminga et al. [57] reported that chondrocytes encapsulated in a fibrin sealant retained their morphology and synthetized matrix in vitro. However, Brittberg et al. suggested that fibrin sealants were not stable for osteochondral healing in vivo [58]. In a second in vitro experiment, chondrocyte migration into the fibrin sealant was evaluated in comparison to the chondrocyte migration into rabbit and human blood clots. Whereas, a migration of chondrocytes into blood clots was observed, no chondrocyte migration into the fibrin sealant occurred [58]. Fbrin sealant contains a high concentration of clotting factors but an insufficient amount of stimulating factors [58]. Furthermore, Cheung et al. suggested that fibrin sealants might impair the migration of chondrocytes via a barrier effect [59]. Similarly, no effect on chondrocyte growth and proliferation by fibrin glue has been observed in other studies [60, 61].

Growth factors have been widely used in neocartilage production in vitro [60]. IGF-1 and 2 promote differentiation of immature chondrocytes [60]. b-FGF exerts mitogenic effects and promotes cell survival [60]. Fibrin glue stabilizes growth factors and other proteins, which prevents natural enzymatic degradation [62]. However, a previous study demonstrated a negative effect of growth factors on the production of neocartilage in vitro [60]. They assumed dose-dependent effects as the in vitro concentrations of growth factors have not been examined [60]. For example, a high concentration of platelets was reported to inhibit proliferation, migration, and the production of collagen type I in human tenocytes [63]. Therefore, the inhibitory effects observed in vitro using high concentrations of fibrin or growth factors strongly suggest impairment of wound healing in vivo.

This study has certainly several limitations. The intraarticular environment is rich of proteins, cells, and cytokines which may influence chondrocytes migration and proliferation. Moreover, the repetitive cycles of weight bearing and motion occurring in the postoperative period have not been considered in the present investigation. Further, the chondrocytes were obtained by only three different donors, which may also not reflect the biological variability of human. Finally, the follow-up was eight weeks, which may limit the capability to identify long-term migration and proliferation of chondrocytes. These limitations should be overcome by future studies, which should also compare the histological changes in vivo of fibrin adduction.

## Conclusion

The use of fibrin glue over a resorbable membrane leads to lower in vitro proliferation and migration of chondrocytes.

## Data Availability

The datasets generated during and/or analysed during the current study are available throughout the manuscript.
