# Correction: Fibrin glue does not assist migration and proliferation of chondrocytes in collagenic membranes: an in vitro study

**DOI:** 10.1186/s13018-022-03370-4

**Published:** 2022-11-05

**Authors:** Filippo Migliorini, Julia Prinz, Nicola Maffulli, Jörg Eschweiler, Christian Weber, Sophie Lecouturier, Frank Hildebrand, Johannes Greven, Hanno Schenker

**Affiliations:** 1 Department of Orthopaedic, Trauma, and Reconstructive Surgery, RWTH University Hospital, Pauwelsstraße 30, 52074 Aachen, Germany; 2 Department of Ophthalmology, RWTH University Hospital, Pauwelsstr. 30, 52074 Aachen, Germany; 3 Department of Medicine, Surgery and Dentistry, University of Salerno, 84081 Baronissi, SA Italy; 4 School of Pharmacy and Bioengineering, Faculty of Medicine, Keele University, ST4 7QB Stoke on Trent, England; 5 Barts and the London School of Medicine and Dentistry, Centre for Sports and Exercise Medicine, Queen Mary University of London, Mile End Hospital, 275 Bancroft Road, E1 4DG London, England

## Correction to: Journal of Orthopaedic Surgery and Research (2022) 17:311 10.1186/s13018-022-03201-6

Following publication of the original article [1], the authors identified an error in the author’s name of Sophie Lecout**u**rier. The correct author’s name is provided in this correction.

An error was identified in the following section,

**Abstract:**

… No difference was found at week **3,** 6, and 8 …

*This should be* … No difference was found at week **4**, 6, and 8 …

**Methods:**

… collagen I/III porcine derived membrane (**Cartmaix**, Matricel GmbH, Herzogenrath, Germany) …

*This should be* … collagen I/III porcine derived membrane (**Cartimaix**, Matricel GmbH, Herzogenrath, Germany)

… the membranes of a density per membrane of approximately 100,000 **MSCs** per cm^2^ …

*This should be* … the membranes of a density per membrane of approximately 100,000 **chondrocytes** per cm^2^

Afterwards, the membranes were dehydrated in **an ascending** alcohol series (5 min per cuvette) as follow: xylene (3×), 100% ethanol (2×), 96% ethanol, 80% ethanol, 70% ethanol, aqua dest. Subsequently the membranes were …

*This should be* … Afterwards, the membranes were dehydrated in **a descending** alcohol series. Subsequently the membranes were

**Results:**

… No difference was found at week **3,** 6, and 8 …

*This should be* … No difference was found at week **4**, 6, and 8 …

Figures 1, 2, 3 are wrong. We provide the corrected Figures 1, 2 and 3

**Fig. 1:**
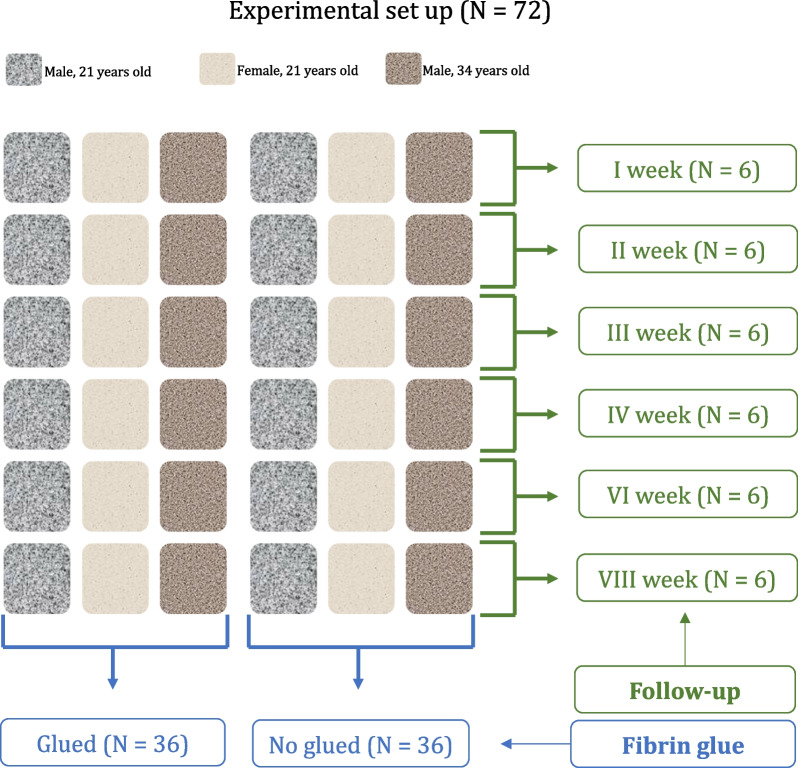
Experimental set-up (*N* = 72)

**Fig. 2:**
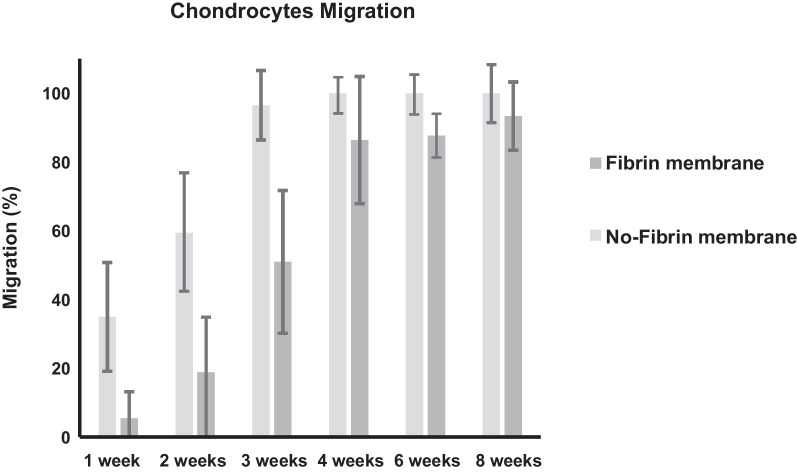
Chondrocytes migration within the membrane

**Fig. 3:**
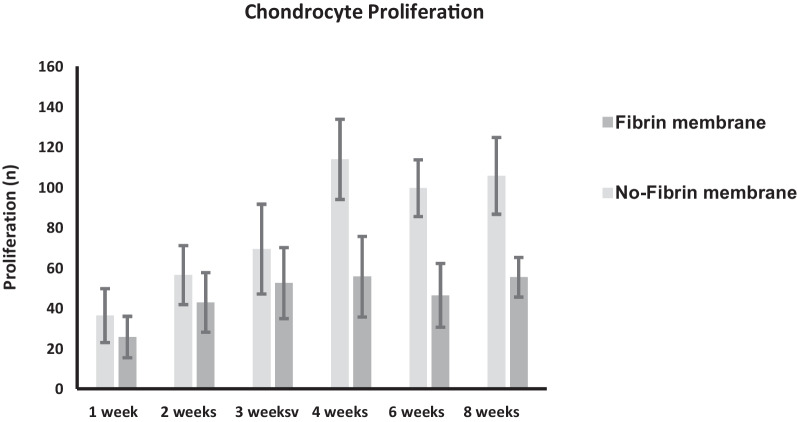
Chondrocytes proliferation within the membrane

## References

[1] Migliorini F, Prinz J, Maffulli N (2022). Fibrin glue does not assist migration and proliferation of chondrocytes in collagenic membranes: an in vitro study. J Orthop Surg Res.

